# Altered reward processing in pathological computer gamers – ERP-results from a semi-natural Gaming-Design

**DOI:** 10.1002/brb3.293

**Published:** 2014-12-23

**Authors:** Eva C P Duven, Kai W Müller, Manfred E Beutel, Klaus Wölfling

**Affiliations:** Outpatient Clinic for Behavioral Addictions, Department of Psychosomatic Medicine and PsychotherapyUniversity Medicine Mainz

**Keywords:** Event-related potentials, incentive sensitization, internet gaming disorder, pathological computer gaming, psychophysiological responses, tolerance

## Abstract

**Introduction:**

Internet Gaming Disorder has been added as a research diagnosis in section III for the DSM-V. Previous findings from neuroscientific research indicate an enhanced motivational attention toward cues related to computer games, similar to findings in substance-related addictions. On the other hand in clinical observational studies tolerance effects are reported by patients with Internet Gaming disorder. In the present study we investigated whether an enhanced motivational attention or tolerance effects are present in patients with Internet Gaming Disorder.

**Methods:**

A clinical sample from the Outpatient Clinic for Behavioral Addictions in Mainz, Germany was recruited, fulfilling the diagnostic criteria for Internet Gaming Disorder. In a semi-natural EEG design participants played a computer game during the recording of event-related potentials to assess reward processing.

**Results:**

The results indicated an attenuated P300 for patients with Internet Gaming Disorder in response to rewards in comparison to healthy controls, while the latency of N100 was prolonged and the amplitude of N100 was increased.

**Conclusions:**

Our findings support the hypothesis that tolerance effects are present in patients with Internet Gaming Disorder, when actively playing computer games. In addition, the initial orienting toward the gaming reward is suggested to consume more capacity for patients with Internet Gaming Disorder, which has been similarly reported by other studies with other methodological background in disorders of substance-related addictions.

## Introduction

The excessive use of applications in the internet, predominantly the use of online games, has been under investigation by a growing number of researchers during the past 10 years. Recently a prevalence of approximately 3.8% in youths (14–24 years of age) has been estimated in Germany (Rumpf et al. 2011), indicating a developing health problem that has a similar impact in other industrial countries (Kuss and Grifffiths 2011). Thus, Internet Gaming Disorder (IGD) has been added as a research diagnosis in section III for the recently published DSM-V (Holden 2010; APA 2012) in the new diagnostic chapter “Substance Use and Addictive Disorders”, while “Gambling Disorder” is proposed to be included in the section as a first behavioral addiction diagnosis. By categorizing the excessive use of the online games as addiction-related, new treatment strategies emerge that closely follow the structure of therapeutic interventions in substance-related disorders, which are currently evaluated (Jäger et al. 2012). Already in 2009 this led to a call for multimodal research projects in behavioral addictions in German psychological and psychosomatic associations (see also Wölfling et al. 2009).

The motivational relevance of addiction-specific cues in the development of addiction has been reported in numerous studies, which focus on how the brain of addicted individuals, regardless of the specific form of dependence, reacts toward those addiction-related cues. This idea is based on the assumption that the brain is not able to process all environmental stimuli simultaneously. Behaviorally important information is therefore selectively attended to, which is thought to be guided by stimulus-related effects but also by properties of the observer himself. Brosch et al. (2011) suggest that different sub processes are acting together to form selective attention. These include networks for endo- and exogenous cues, but also special processes for emotionally salient cues (Lang et al. 1997; Compton 2003; Vuilleumier 2005; Williams and Gordon 2007). Selective attention toward these cues is thought to be unconscious, however, influenced by current emotional states (e.g. Fox et al. 2001; Bishop et al. 2004, 2007). The development of unconscious selective attention toward behaviorally relevant cues in addiction is described by the theoretical framework of incentive sensitization by Robinson and Berridge (2003) as increased “wanting” of the drug/behavior through an alteration of the brain reward system is basic to cue-reactivity paradigms. These paradigms assess how the brain reacts to cues that have become motivationally relevant to the addicted individual.

Thalemann et al. (2007) investigated specific cue-reactivity to computer game-related cues in excessive computer gamers by using electroencephalography (EEG). They compared the reactions of excessive (gamers meeting at least three criteria for IGD) and casual computer gamers to computer game-relevant and -irrelevant cues to find out whether emotional processing differed between the groups. The excessive computer gamers were found to have an increased response to the computer game-relevant cues in comparison to the casual gamers who responded to the computer game-relevant cues in the same way as to the neutral cues. An enhanced late positive complex (LPC) which seems to be functionally similar to the P300 component in the ERP (Kok 1997) was recorded for the excessive computer gamers as a reaction to the computer game-relevant cues. Casual gamers displayed an enhanced LPC only for cues of emotional valence (positive and negative). This pattern is similar to reactions of substance-dependent subjects to substance-relevant cues (Herrmann et al. 2000; Franken et al. 2003; Namkoong et al. 2004; Wölfling et al. 2008; Dunning et al. 2011) and of pathological gamblers to gambling-cues (Wölfling et al. 2011a). The authors interpreted these findings as support for an altered motivational processing in excessive computer gaming, consistent with prior studies on cue reactivity in substance dependence (Grüsser et al. 2004; Dunning et al. 2011). A meta-analysis of the electrophysiological indices of biased cognitive processing of substance-related cues by Littel et al. indicated that the P300 component of the ERP to substance cues was generally larger in individuals with substance use disorders than in healthy controls (Littel et al. 2012). The P300 component is a large positive component that usually occurs 300–800 ms after the presentation of the stimulus. It has been extensively shown that it is triggered by infrequent stimuli, for example, in an oddball paradigm (Donchin 1981) and seems to be related to the deployment of attentional resources to task-relevant stimuli. In addiction stimuli elicit a larger P300 are expected to have become motivationally relevant. Likewise, in an fMRI-study Ko et al. (2011) found that cue-exposure of online-game screenshots led to increased activation of bilateral dorsolateral prefrontal cortex (DLPFC) in subjects meeting criteria for Internet Gaming Disorder, reflecting enhanced emotion processing. In adolescents who frequently played video games higher left ventral striatum volume was found with voxel-based morphometry (VBM), which is thought to reflect altered reward processing and represent adaptive neural plasticity (Kühn et al. 2011). General similarities in the underlying brain processes involved in abuse—whether substance dependent or behavioral—have been shown (for reviews see Frascella et al. 2010; Yuan et al. 2011).

Similar to the tolerance in other forms of dependencies an increase of time or in frequency of computer gaming can be observed in IGD. In an epidemiological study Batthyány et al. (2009) found that students who fulfilled the criteria of Internet Gaming Disorder measured by the AICA-S self-report scale (Scale for the Assessment of Internet and Computer game Addiction (Wölfling et al. 2011b)) also reported a more pronounced increase in the amount of time spent playing computer games (Batthyány et al. 2009). Tolerance is thought to develop through neurobiological processes of adaptation leading to habituation of the individual reaction toward the addictive substance or a specific behavior. As opposed to the model of incentive sensitization, the concept of tolerance describes the process that increases the necessity for dependent individuals to seek the drug/behavior in higher doses or increased frequency. The individual becomes habituated to the effects of the drug or behavior itself, not to the cues or the visual/auditory presentation of the drug/behavior as in incentive sensitization. Individuals who abuse computer gaming therefore need to play more often or for a longer duration to achieve the same effects (e.g. stimulation) as before which also can be explained by an altered reward system in the brain. Neurotransmitter-systems involved in the perception of reward (Serotonin, Dopamine) seem to be disturbed not only in drug-related but also in behavioral addiction, as shown by Hu and collogues who used single photoemission computed tomography (SPECT) to investigate whether striatal dopamine transporter (DAT) levels were altered in individuals with IGD and found supporting results (Hou et al. 2012).

Usually it is suggested that individuals prone to dependence have a reduced sensitivity toward natural rewards, leading them to seek out stronger rewards by using drugs or excessive behaviors (Griffiths 2005; Volkow et al. 2011). The recurrent use of the drug further diminishes the reward sensitivity toward dependence cues as the body reacts toward cues signalizing drug intake or exertion of excessive behavior. Specifically opponent-processes are initiated to maintain a form of homeostasis. This repeated alteration of the reward system in reaction to recurrent drug intake or exertion of excessive behavior can lead to a state of allostasis (chronic deviation of reward set point). This might lead to compensatory processes in other systems—for example, in a constantly reduced sensitivity to natural rewards (Koob and Le Moal 2001). In substance use disorders (SUD's), such as nicotine, alcohol, opioid, and cocaine dependence a generally reduced P300 in reaction in cognitive tasks has been shown (Anokhin et al. 2000; Bauer 2001; Papageorgiou et al. 2004; Fein and Chang 2006; Gooding et al. 2008). However, a study by Parvaz et al. (2012) was able to show that the modulation of the P300 by reward value was blunted in individuals with current or recent cocaine use disorder, leading to the assumption that sensitivity to reward in SUD's can be operationalized via an attenuated amplitude of the P300 component in the ERP to rewards. In IGD, individuals are constantly rewarding themselves by the use of a highly rewarding environment. Rewards are manifold in these environments, ranging from simple intermitted reinforcement by completing simple tasks as gathering a certain amount of tokens to social reinforcement by overall social prestige in a group of players. Other natural reinforcers such as food or sex become less rewarding during tolerance processes as the general sensitivity to rewards is reduced. This in turn can lead to a total retreat from natural reinforcers in the real world. However, a higher sensitivity to rewards for individuals with IGD has been found in two studies in Asia (Dong et al. 2011; Yen et al. 2012). Yen et al. (2012) investigated whether the responsiveness to rewards (in this case operationalized via the behavioral activation system) changed over the course of 1 year in adolescents showing IGD. Indeed, even though the initial responsiveness to reward was higher in adolescents with IGD it became lower than in the control group after 1 year. The authors suggested that this might be a development within the course of IGD that leads to less offline-interaction. In general there seem to be conflicting results on this matter for IGD.

Anhedonia, the downside of the habituation to the drug/excessive behavior has not been investigated yet for IGD. In clinical interviews patients report having less pleasure in activities previously found rewarding (Beutel et al. 2011), which is in line with current frameworks on the role of anhedonia in addiction in general, including substance addictions (Wise 2008). In order to investigate the specificity of cue-reactivity in pathological computer gamers we think it is necessary to assess whether rewards in computer games in general elicit enhanced motivational attention in the sense of action orienting (see also Balcetis and Dunning (2006) for motivational attention) or whether tolerance effects in accordance to reduced sensitivity to specific computer game rewards are visible. For this purpose a semi-natural design in which players can retrieve rewards might be suitable.

The present study uses a semi-natural design in patients suffering from IGD. The task consists of a computer game where the participants have to acquire tokens by navigating through a virtual environment while high-resolution EEG signal is recorded. With comparison to visual addiction-related cues triggering craving, the processing of reward might be better operationalized by the ERP component P300, reflecting attention and outcome evaluation. It has been reliably shown that the amplitude of the P300 is affected by reward magnitude (Wu and Zhou 2009) in healthy subjects, with higher amplitude in conditions with higher rewards compared to lower rewards in simple card-playing games. In these tasks P300 in reward processing is constantly assessed during feedback after each trial when the participant is informed whether or not he won money. In the study of semi-natural computer game-rewards the P300 is therefore the component of interest, as we are interested in the finding and perception of reward. To also investigate potential changes in attentional processing between the groups components prior to the P300 are also assessed. The main goal of the present study is to explore whether it is possible to identify differences between pathological computer game players and casual players on the cortical level. The semi-natural setting gives the opportunity to record EEG in an environment that is close to the usually environment of pathological computer gamers. Specifically we want to explore whether pathological computer gamers show tolerance effects as evidenced by a reduced P300 in the ERP or whether motivational attention is enhanced in reaction to finding a reward as evidenced by increased amplitude of the P300 and later components.

## Methods

### Sample

The sample originally consisted of 30 male participants, of whom 15 fulfilled the criteria for IGD, especially pathological computer gaming (PCG), and 15 fulfilled the criteria of casual computer gaming (CG). This classification was made with the self-report questionnaire Scale for the Assessment of Internet and Computer game Addiction (AICA-S, CSV-S (Wölfling et al. 2011b)). A cutoff score of seven of a possible total score of 27 is an indicator for addicted computer gaming. Casual computer gaming is classified as a score of 0–3 (Wölfling et al. 2011b). Additionally, the participants classified as pathological computer gamers underwent a clinical interview by an expert psychologist in order to establish need for treatment. The clinical sample was recruited via the Outpatient Clinic for Behavioral Addictions, University Medical Center Mainz, an outpatient clinic for the treatment of Internet Addiction (Beutel et al. 2011) CG were recruited via the Johannes Gutenberg-University Mainz. For EEG-measurement purposes only right-handed participants of full age with normal or corrected-to-normal vision were included. In the EEG-analysis a total of three subjects were excluded from the analysis due to technical problems (*n* = 1) and due to bad data quality (*n* = 2), leaving 27 participants (PCG *n* = 14; CG *n* = 13; for details of social demographics see Table 1).

**Table 1:** Social demographics

	PCG (*N* = 14) M (SD)	CG (*N* = 13) M (SD)	*t* (df)	Sig.
Age	24.29 (5.84)	23.31 (3.01)	−0.541 (25)	*P* = 0.59
	% (*n*)	% (*n*)	Chi^2^ (df)	Sig.
Male	100 (14)	100 (13)		
Marital status (single)	92.86 (13)	100 (13)	0.964 (1)	ns
Qualification for higher education	69.23 (9)	100 (13)	3.134 (1)	ns
Employment/in training (yes)	71.43 (10)	100 (13)	4.360 (1)	*P* ≤ 0.05

PCG, Pathological Computer Gamers; CG, Casual Gamers; ns, nonsignificant.

### Measurements

#### Aica-s (Wölfling et al. 2011b)

The AICA-S assesses the amount of computer gaming with two open items (“How many hours do you play on average during the weekend/holidays”, translated from the German version), two closed items on the amount of computer gaming, ten Likert-scale items as, for example, “How often do you play computer games, even though you planned not to play or played more often or longer than you actually planned” (translated from the German version), which can be answered on a five-point likert-scale (“0” = “never”, “4” = always), and one closed item on problems related to the computer gaming. Its items are closely related to clinical criteria of substance-related addictions and thus operationalize IGD in a similar way as the Internet Addiction Test (Young 2007), a wide spread measure for IGD. Reliability and factorial as well as construct validity of AICA-S was well demonstrated (Wölfling et al. 2011b).

#### Scl-90-r (Franke 2002)

The SCL-90R is a 90-item, self-reported inventory widely used to measure current distress. Nine separate syndrome scales can be calculated. Various studies have proven the validity of the scales (Franke 1995; Rief et al. 2001). The reliability (internal consistency) of the nine scales lies between 0.80 and 0.90. The scale for depressiveness is used here, as group differences on depression may have influence on the P300 (Singh et al. 2000).

#### Semi-natural design (2D-Computer Game sequence)

The computer game sequence used in this experiment consisted of a specifically programmed virtual environment through which the participants had to maneuver to acquire tokens (small trophy symbols). None of the participants had seen or played this specific computer game sequence before. The virtual environment was programmed in 2D and consisted of an 18.5 cm*24.5 cm maze in which 20 tokens were hidden. Those tokens were only visible when the player had navigated to a point, at which it was physically possible to see the token (e.g., it is impossible to see a token though a wall). The token stayed visible until it was collected. When the token was collected a short sound occurred. To navigate around the environment players had to press the buttons A, S, D, and W on a German keyboard, as those buttons are used in the game which most of the pathological gamers play (e.g., World of Warcraft). The frequency in which the tokens were collected was depending on the players decision where to navigate the counter. The 2D Game was designed to be not generally arousing so that the occurrence of reward would stand out and overall arousal would not confound the effects. All participants finished the task successfully. Triggers were sent to Analyzer Software whenever a token was visible and whenever it was acquired. The whole computer game sequence was presented using Presentation® software (Version 9, www.neurobs.com).

### Procedure

The pathological computer game players were asked for participation during the intake interview of the Outpatient Clinic for Behavioral Addictions. The casual computer gamers were recruited using leaflets at the Johannes Gutenberg-University. Both groups were screened for IGD with the AICA-S (Wölfling et al. 2011b) and included into the study when fulfilling the inclusion criteria. They were then invited to the EEG-laboratory and signed informed consent. The electrode cap used was applied to the participants head and the peripheral electrodes were placed. The task of the participant was to acquire as many tokens spread over the virtual environment as possible within 20 min. After the experiment the monetary reward was handed out.

### Apparatus

Electroencephalography (EEG) was recorded using the international EEG 10–20 system placement with 61 electrodes. Eye movement (horizontal ocular movements and eye blinks) were recorded from peripheral electrodes placed at the outer canti of both eyes (HEOG) as well as above and below the left eye (VEOG). All electrodes were referenced to linked mastoids during the recording. Impedance thresholds of all electrodes were kept below 10 kΩ.

### Design

The EEG-data were recorded using Brain Vision Recorder software and analyzed using Brain Vision Analyzer 2.0.1 software (Brain Products, 2012) to identify event-related potentials (ERP's). We applied the Gratton & Coles occular correction algorithm and filtered the data (low cutoff at 1 Hz, high cutoff at 40 Hz, notch at 50 Hz). The data were then segmented to epochs of 1200 ms (−200 ms to 1000 ms) by markers set by the Presentation software (Neurobehavioral Systems) around finding the reward and semiautomatically checked for artifacts. In total only 2.96% of the epochs were rejected and there was no significant difference for the number of rejected epochs between the groups (*T* = 1.540, *P* = 0.136). Nine channels were used for ERP analysis (sagittal: left hemisphere (F3, C3, P3), central (Fz, Cz, Pz), right hemisphere (F4, C4, P4); coronal: frontal (F3, Fz, F4), central (C3, Cz, C4), parietal (P3, Pz, P4)) similar to Wölfling et al. (2008). A more extensive analysis of all electrodes revealed similar results, however, only those for nine channels are reported for parsimony. After visual inspection peak detection was used to detect differences between peaks of the N100, N200, P200, P300 and the results were exported per electrode and participant to IBM SPSS Statistics Version 20. The time range for the different components were 100 ms to 180 ms for N100, for N200 180–300 ms, for P200 150 ms to 250 ms and for P300 200 ms to 600 ms. For the ERP-data separate 2 × 3 × 3 repeated-measures ANOVAs with group as between subject factor (PCG vs. CG) and sagittal (left, central, right) and coronal (frontal, central, parietal) electrode position as within-subject factors were conducted. Where the sphericity assumption was violated, Greenhouse-Geisser-Corrections were applied; otherwise Pillai-Spur is reported. Significant interactions are followed up by Bonferroni-corrected post-hoc analyses.

### Ethics statement

The conducted study was performed in accordance to the declaration of Helsinki and approved by the local ethical commission of the medical chamber of Rhineland-Palatinate, a federal state of Germany. All participants received a monetary reward for participation, were informed about the study's purpose and asked to give oral and written informed consent.

## Results

As expected, pathological computer gamers scored significantly higher on the AICA-S (Wölfling et al. 2011b) than the control group. They also reported longer hours of playtime during the week and on the weekend and a higher number of negative consequences resulting from playing computer games. In addition, they also reported higher craving and cognitive involvement with computer games (see Table 2). However, the groups did not differ on ratings for depressiveness, as measured with subscale from the SCL-90R (Franke 2002).

**Table 2:** Depressiveness and Computer game playing behavior

	Pathological Computer Gamers (*N* = 14) Mean (SD)	Casual Gamers (*N* = 13) Mean (SD)	*F* (df)	Sig.
SCL-Depressiveness-Subscale	0.43 (0.51)	0.15 (0.38)	2.48 (1)	ns
AICA-S Score^1^ (Wölfling et al. [Bibr b56][Bibr b57])	13.89 (3.85)	0.85 (0.83)	142.87 (1)	*P* ≤ 0.001
h/weekday playtime	7.18 (3.29)	1.08 (1.08)	40.44 (1)	*P* ≤ 0.001
h/holiday playtime	9.04 (3.69)	1.92 (1.30)	43.13 (1)	*P* ≤ 0.001
Number of negative consequences^2^	3.79 (1.72)	0.77 (0.93)	31.51 (1)	*P* ≤ 0.001
Craving to play computer games^3^	2.43 (1.22)	0.23 (0.44)	63.08 (1)	*P* ≤ 0.001
Cognitive involvement with computer games^3^	2.71 (0.83)	0.85 (0.56)	196.56 (1)	*P* ≤ 0.001

AICA-S, Assessment of Internet and Computer game Addiction.

1 AICA-S Score (0–27), 13.5 is cutoff for addiction.

2 in consequence of computer game playing.

3 Item from AICA-S, Likert-Scale from 0 to 4 (from “not at all” to “very strong”).

For EEG-Data only significant group and group-interaction effects are reported.

### Latencies

The latencies of the components were entered into a MANOVA, revealing significant differences between PCG and CG for the N100 (*F*_1_ = 5.728, *P* = 0.028) with longer latencies for PCG.

### Peak amplitudes

**Figure 1:**
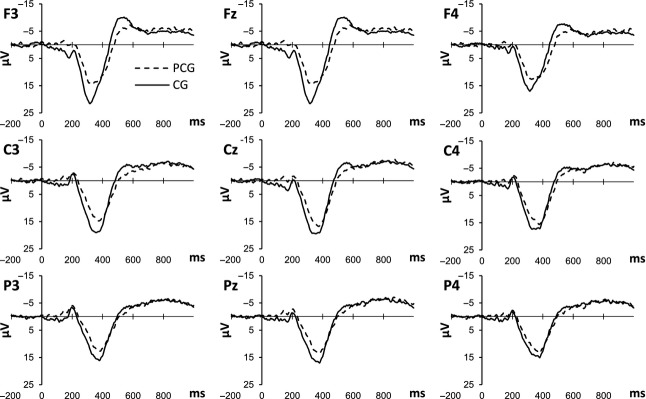
Grand averaged ERP (*μ*V) elicited by finding a reward in PCG (Pathological Computer Gamers) and CG (Casual Gamers).

**Figure 2:**
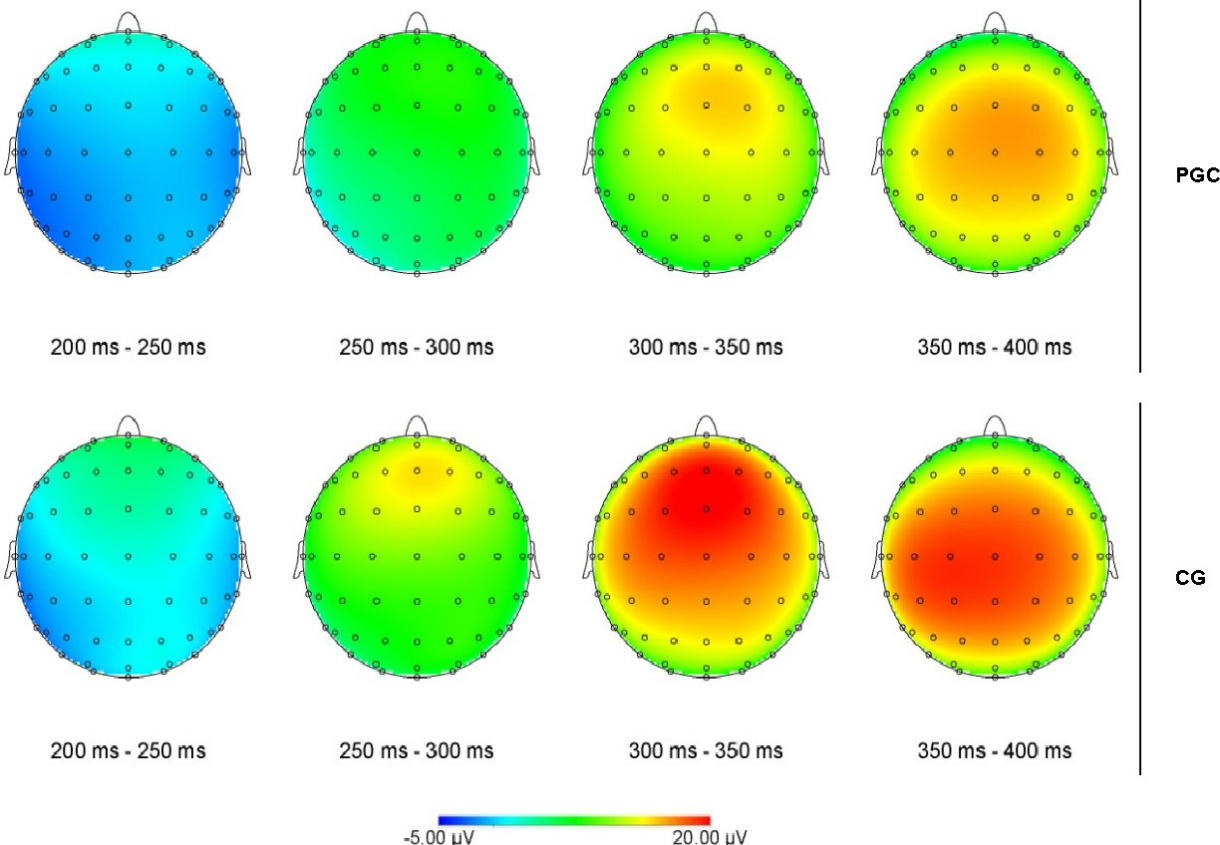
Scalp topography of the event of finding a reward by PGC (Pathological Computer Gamers) and CG (Casual Gamers) in the time window from 200 to 400 ms.

The amplitude of the N100 was qualified by a significant interaction between sagittal electrode position and group (*F*_2_ = 13.55, *P* < 0.001). Post-hoc analyses showed that PCG had a significantly more negative N100 amplitude in comparison to CG on the left hemisphere (*F* = 7.452, *P* = 0.011) and on midline (*F*_1_ = 14.54, *P* = 0.001). For N200 peak amplitude sagittal electrode position significantly interacted with group (*F*_1_ = 10.633, *P* < 0.001), however, there were no significant between-groups effects. A significant interaction between sagittal electrode position and group was found for the P200 (*F*_2_ = 5.036, *P* = 0.015). Post-hoc analyses showed that PCG had a significantly less positive P200 amplitude in comparison to CG on the left hemisphere (*F*_1_ = 4.950, *P* = 0.035) and on midline (*F*_1_ = 7.997, *P* = 0.009). For the P300 peak there was a significant interaction between sagittal electrode position and group (*F*_2_ = 5.576, *P* = 0.01; see Fig.1). Post-hoc analyses showed that PCG had a significantly less positive P300 amplitude in comparison to CG on the left hemisphere (*F*_1_ = 7.403, *P* = 0.012) and on midline (*F*_1_ = 6.738, *P* = 0.016; see Fig.2 and Table 3). In summary PCG showed longer latencies for the N100 and enhanced N100 and reduced P200 and P300 amplitudes especially on the left hemisphere and on central electrode sites. Significant results for N200 amplitude were based on differences within groups.

**Table 3:** Mean latencies and amplitudes for N100, N200, P200, and P300

		Pathological Computer Gamers (*N* = 14) Mean (SD)	Casual Gamers (*N* = 13) Mean (SD)	*F* (df)	Sig	Direction of effect
N100	Latency (ms)	151.00 (17.16)	128.92 (30.69)	5.428 (1)	*P* = 0.028	PCG > CG
	Right (*μ*V)	−2.60 (2.28)	−1.17 (1.46)	3.705 (1)	ns	–
	Midline (*μ*V)	−3.22 (2.24)	−0.40 (1.50)	14.542 (1)	*P* = 0.001	PCG > CG
	Left (*μ*V)	−3.52 (2.29)	−1.36 (1.77)	7.452 (1)	*P* = 0.011	PCG > CG
N200	Latency (ms)	197.14 (19.89)	204.31 (20.05)	0.868 (1)	ns	–
	Right (*μ*V)	−2.91 (3.20)	−2.01 (3.75)	0.448 (1)	ns	–
	Midline (*μ*V)	−3.17 (3.53)	−1.27 (3.88)	1.787 (1)	ns	–
	Left (*μ*V)	−3.98 (3.23)	−3.25 (3.58)	0.312 (1)	ns	–
P200	Latency (ms)	223.43 (30.15)	223.23 (28.93)	0.000 (1)	ns	–
	Right (*μ*V)	4.39 (2.88)	6.52 (3.49)	3.023 (1)	ns	–
	Midline (*μ*V)	4.14 (3.28)	7.66 (3.18)	7.997 (1)	*P* = 0.009	PCG < CG
	Left (*μ*V)	3.21 (3.21)	6.13 (3.62)	4.950 (1)	*P* = 0.035	PCG < CG
P300	Latency (ms)	344.43 (59.01)	317.38 (35.59)	2.038 (1)	ns	–
	Right (*μ*V)	16.03 (4.55)	19.12 (3.96)	3.526 (1)	ns	–
	Midline (*μ*V)	17.12 (4.76)	22.06 (5.12)	6.738 (1)	*P* = 0.016	PCG < CG
	Left (*μ*V)	15.20 (3.98)	19.56 (4.34)	7.403 (1)	*P* = 0.012	PCG < CG

PCG, pathological computer gamers; CG, casual gamers; ns, nonsignificant.

Mean latencies (ms) and amplitudes (*μ*V) with standard deviations.

## Discussion

The aim of the present study was to identify an altered mechanism of reward processing in IGD. Therefore, differences between pathological computer game players and casual players on an electrophysiological level were expected. For this purpose a semi-natural gaming sequence was programmed in which pathological computer gamers had to acquire tokens by navigating playfully through a virtual environment. Specifically we wanted to explore whether an enhanced motivational attention or tolerance was present during the finding of rewards. The results indicated significant differences in mean amplitude of the P300 between the groups during the finding of reward. There are two possible explanations four our findings. First, individuals with IGD might have a general reduced sensitivity to “natural rewards”. This argumentation is especially interesting as previous research in cognitive processing in substance-dependent individuals attributed the attenuated amplitude of the P300 to the effects of the substances on the brain's reward circuits. As the blunted reaction toward rewards has been shown by Parvaz et al. and as in IGD there is no substance involved this explanation would definitely support the idea that other processes than the detrimental effects of the drug are involved in how the reward sensitivity of the brain is changed, namely underlying cognitive and emotional dysfunction in addiction (Parvaz et al. 2012). This explanation is difficult as a previous study on the sensitivity to rewards by Dong et al. (2011) found a hypersensitivity to guessing-game rewards in IGD, which is in conflict with the current findings. In addition the group of IGD is special in the sense that in contrary to, for example, guessing games in SUD's the computer game reward actually is was constitutes the rewarding effect of the “drug” computer game. Therefore, a second explanation is possible, namely that our findings support the idea that the electrophysiological reaction toward rewards is a correlate of opponent-processes leading to tolerance (Koob and Le Moal 2001), a well-described brain reward circuit alteration in SUD's. According to Volkow et al. (2010) there is a difference between the sensitivity of reward circuits (lowered in individuals with SUDs and leading to a lower “high”) and an enhanced sensitivity to conditioned expectations to drug effects, that is based on the enhanced memory circuits over the development of addiction disorders. Our method investigated the direct response to the reward, not the expectancy of the reward and might therefore especially tap the sensitivity of the reward circuits of patients suffering from IGD.

For a more detailed analysis of the findings the differences between the groups on earlier components seem relevant. Pathological computer gamers had slightly longer latencies for the N100, which was also more negative in amplitude. The N100 is typically generated by perception of stimuli and especially by audiovisual stimuli as in this experiment. Previous research has shown that shorter peak latencies and smaller amplitudes reflect facilitated processing of stimuli by multimodal presentation (Van Wassenhove et al. 2005; Stekelenburg and Vroomen 2007). Also the N100 is suggested to be enhanced by attention toward the stimulus (Van der Lubbe et al. 2011). Therefore, the shorter latency and smaller peak in the group of casual gamers might be due to pathological computer gamers initially needing more capacity to process the gaming reward, while later evaluation of the reward demands less attention, as reflected by reduced P300 amplitude. A probable explanation would be that pathological computer gamers showed an initial orienting toward the gaming reward, which is often found in dependent individuals toward drug-related cues. Especially when individuals are already in the maintenance phase of addiction—which is the case in the present sample—habituation processes might lead to less attentional processing of addiction-related cues, while initial orienting remains unaffected (Vollstadt-Klein et al. 2009). This could lead to an increased risk for relapse, for example, in anxiety disorders initial orienting in attentional biases is thought to persist after treatment (Koster et al. 2010). Also a reduced P300 has been reported to probably stem from reduced motivation (Kleih et al. 2010) or from less perceived magnitude of reward (Beutel et al. 2011). In general this might reflect the decreased sensitivity of the brain reward system due to overuse and learning processes, leading to tolerance and less attentional motivation toward rewards (Wu and Zhou 2009). Generally this theoretical approach is in line with a more habit-based form of learning during the development of addiction, as proposed by Everitt and Robbins (2005). To their mind stimulus-response patterns are formed as cues repeatedly lead to drug-taking behavior, decreasing incentive sensitization processes. These are reactivated, however, when drug-taking is prohibited or unavailable. In our paradigm participants were free to play and might therefore have shown a general habit-based reaction toward the gaming rewards.

This study was conducted as an attempt to narrow down the suggested altered mechanisms of the brain reward system of this new and emerging disorder. The design is exploratory in investigating a semi-natural design that enables the comparison of individuals' performance and electrophysiology in a standardized way. The exploratory design naturally has some limitations. Due to the semi-natural design we did not control for motor activation. This could have led to differences in motor activity and motor skills between the groups which might have had influence on the P300 amplitudes. However, the casual gamers also had extensive experience with computer games and appeared to be equally skilled in typing. Also there was a large heterogeneity in gaming hours per day within the group of pathological computer gamers, with some already being abstinent or almost abstinent. This might have led to smaller effect sizes. In addition, we did not control for arousal and vigilance during the task. Probably the participants with IGD were less aroused by the 2D-Computer Game than the control group. However, decreased arousal in reaction to reward in Computer Games might be directly related to tolerance effects in IGD. Nonetheless future studies should aim at controlling for arousal during the measurement. Most importantly future studies should compare the reaction of individuals with IGD to computer game rewards directly to other rewards such as monetary rewards in guessing games to identify differences in general reward sensitivity and specific tolerance effects to the rewarding effect of the “drug” (computer games).

In summary, we conclude that pathological computer gamers indeed show a divergent processing of reward stimuli within the semi-natural design. Following up the present data we would suggest controlling for motor skills and including parameters of how pleasant the rewards were perceived and on motivation for the task. With the new research diagnosis at hand future studies should also include patients with more severe IGD than in outpatient settings for greater effect sizes (for example from inpatient settings). Investigating the effects of reward within paradigms that are as natural as possible and that stay close to the reality of the participants is essential in further understanding the symptom complex of Internet Gaming Disorder. In general our data suggest that also in behavioral addiction opponent-processes in reaction to the rewarding “excessive behavior effect” (similar to the expression “drug effect”) can be found.
